# In Situ Formed Composite Polymer Electrolytes Based on Anion‐Trapping Boron Moiety and Polyhedral Oligomeric Silsesquioxane for High Performance Lithium Metal Batteries

**DOI:** 10.1002/smsc.202400183

**Published:** 2024-08-12

**Authors:** Chia‐Chi Chang, Min‐Hsien Shen, Yuan‐Shuo Hsu, Hsisheng Teng, Jeng‐Shiung Jan

**Affiliations:** ^1^ Department of Chemical Engineering National Cheng Kung University No. 1 University Road Tainan 70101 Taiwan; ^2^ Hierarchical Green‐Energy Materials (Hi‐GEM) Research Center National Cheng Kung University Tainan 70101 Taiwan; ^3^ Program on Smart and Sustainable Manufacturing Academy of Innovative Semiconductor and Sustainable Manufacturing National Cheng Kung University Tainan 70101 Taiwan

**Keywords:** boron moiety, composite polymer electrolyte, in situ polymerization, lithium‐ion battery, POSS

## Abstract

Quasi‐solid and composite polymer electrolytes (QSPEs and CPEs) used in lithium‐ion battery (LIB) have recently been a novel strategy owing to their high‐safety comparing to traditional liquid counterparts. This study reported the preparation of CPEs based on boron moiety, poly(ethylene glycol) (PEG), and octahedral polyhedral oligomeric silsesquioxane (POSS) via in situ thermal polymerization method directly onto the lithium anode to improve the interfacial contact and electrochemical performance. The synergistic effect between the incorporation of anion‐trapping boron moiety and in situ polymerization rendered the QSPEs exhibiting higher electrochemical voltage window, ionic conductivity, and transference number as well as better electrochemical performance than the PEG‐based counterpart. Due to the Lewis acid effect, anion‐trapping boron moiety could promote the dissociation of lithium salts, allowing more lithium ions to be in the free state, thereby enhancing the lithium‐ion conductivity. With an optimal addition of POSS, the as‐prepared CPEs exhibited lower overpotential during the lithium plating‐stripping test and better electrochemical performance than the QSPE counterparts. The optimal POSS addition could facilitate the lithium‐ion conduction and establishment of continuous ion pathways, further improving their electrochemical performance. This study pointed a promising approach for developing high performance lithium‐ion batteries.

## Graphical abstract

The QSPEs containing boron moiety exhibit better electrochemical performance than the PEG‐based counterpart due to the synergistic effect between the incorporation of anion‐trapping boron moiety and in situ polymerization. The optimal POSS addition facilitates the lithium‐ion conduction and establishment of continuous ion pathways, further improving the electrochemical performance of the as‐prepared CPEs.

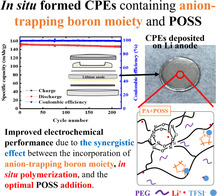

## 1 Introduction

In recent years, with the rapid growth of industry, the combustion of fossil fuels has resulted in significant carbon dioxide emissions, leading to increasingly severe global warming. This has highlighted the importance of developing alternative energy sources. Additionally, rapid technological advancements have led to a growing demand for technology products, thereby increasing the need for efficient and high‐power energy storage methods. Batteries, as energy storage devices, have been in development for many years and included various types such as fuel cells, lead‐acid batteries, and nickel‐cadmium batteries.^[^ ^1^ ^]^ However, these traditional battery technologies have their drawbacks, including large size, heavy weight, lack of portability, battery memory effects, and environmental pollution concerns. In contrast, lithium‐ion batteries (LIBs), as a type of rechargeable secondary battery, offer numerous advantages such as high operating voltage, high energy density, long cycle life, fast charge and discharge capabilities, and minimal environmental impact.^[^ ^2^, ^3^, ^4^, ^5^ ^]^ Due to these advantages, LIBs have widespread applications, from small devices like smartphones, cameras, and medical devices to large‐scale uses such as electric vehicles and submarines.

The working principle of LIBs is based on the movement of lithium ions between the electrodes to store and release energy.^[^ ^6^ ^]^ The electrolyte in a LIB primarily functions to impede the direct flow of electrons while allowing ions to pass through as a medium for the movement of lithium ions between the electrodes. A good electrolyte should be compatible with both the positive and negative electrodes, facilitating lithium‐ion conduction while remaining non‐conductive to electrons. Currently, the primary electrolyte used in LIBs is in liquid form, typically organic solutions containing lithium salts. Traditional liquid electrolytes offer good ionic conductivity and solubility but are susceptible to thermal runaway reactions at high temperatures.^[^ ^7^ ^]^ Moreover, it also comes with limitation such as leakage issues, volatility, and safety concerns. As a result, researchers have turned their attention to the study of solid‐state electrolyte materials with excellent mechanical strength and stability to address these issues.^[^ ^6^, ^8^, ^9^, ^10^ ^]^ However, solid‐state electrolytes face certain challenges including lower ion conductivity compared to liquid electrolytes and the compatibility issues between the electrolyte and electrode interface, which are crucial factors affecting battery performance. Quasi‐solid and composite polymer electrolytes (QSPEs and CPEs) exhibiting the advantage of easy processing could circumvent these drawbacks, making them more promising candidates in practical applications. Poly(ethylene glycol) (PEG) could dissolve in various salts to form ionic‐conductive solutions via interacting with cations, making it the most studied electrolyte in LIBs.^[^ ^11^, ^12^ ^]^ However, there are some drawbacks including the crystallization issue and low Li‐ion transference number (0.2–0.3).^[^ ^13^ ^]^

Recently, boron‐based materials have attracted intensive attention in research related to LIBs due to the unique electronic structures and hybrid forms of boron as well as their tunable physico/chemical and electrochemical properties.^[^ ^14^, ^15^, ^16^, ^17^, ^18^ ^]^ In the past decade, QSPEs and CPEs containing anion‐trapping boron moieties have been investigated in LIBs.^[^ ^14^, ^16^, ^17^, ^18^, ^19^, ^20^, ^21^, ^22^, ^23^ ^]^ The boron moieties in PEs could effectively trap anions through the Lewis acid−base interactions between the boron moieties with empty p orbital and anions, promoting salt dissociation and uniform deposition of metal cations at the anode and stabilizing the anode/electrolyte interface due to the reduced concentration polarization. Herein, this study reported the preparation of QSPEs and CPEs based on *sp* ^2^ boron moiety, poly(ethylene glycol) (PEG), and octahedral polyhedral oligomeric silsesquioxane (POSS) via in situ thermal polymerization method. Our group and others have demonstrated that the in situ polymerization method, which involves thermally polymerizing the electrolyte directly on the lithium anode. This approach could result in the PE being tightly integrated with the electrode, reducing the interface impedance between the electrode and electrolyte.^[^ ^19^, ^24^, ^25^, ^26^ ^]^ The incorporation of *sp* ^2^ boron moiety could facilitate the formation of electron‐deficient covalent compounds, imparting Lewis acid characteristics to the material.^[^ ^14^, ^27^, ^28^, ^29^ ^]^ This anion‐trapping property enables the promotion of a higher degree of lithium ions at ionized state by assisting their dissociation, which, consequently, enhances the lithium‐ion conductivity, establishes continuous ion pathways, and thus improves their electrochemical performance.^[^ ^14^, ^30^, ^31^ ^]^ PEG was employed to serve dual purposes: improving the mechanical properties of the PE and utilizing the polyether segments within its structure to facilitate lithium‐ion conduction through a hopping mechanism.^[^ ^32^ ^]^ Moreover, POSS, consisting of core framework of inorganic Si—O—Si bonds and eight organic groups (R) attached to its periphery, was incorporated in the crosslinked network to form CPEs, which would possess characteristics of both organic materials and inorganic, cage‐like molecular structure, thereby combining the advantages of both.^[^ ^33^, ^34^ ^]^ The presence of oxygen atoms in its structure could facilitate lithium‐ion conduction and the polyhedral structure of POSS could establish continuous ion pathways, enhance lithium‐ion conductivity and, in turn, improve the electrochemical performance of CPEs.^[^ ^35^, ^36^ ^]^ It is anticipated that the synergistic effect between the in situ polymerization and incorporation of anion‐trapping boron moiety and POSS could render the as‐prepared QSPEs and CPEs in LIBs exhibit improved electrochemical properties.

## 2 Experimental Section

### 2.1 Materials

Polyvinylidene fluoride (PVDF, 1 090 000 g mol^−1^, Arkema), carbon black (Super‐P, Timcal), lithium iron phosphate (LFP, Aleees), and N‐methyl‐2‐pyrrolidone (NMP, 99%, Macron) were used without additional purification. 2,2’‐Azobis(2‐methylpropionitrile) (AIBN, Aencore) was purified by recrystallization. Lithium bis(trifluoromethanesulfonyl) imide (LiTFSI, 99.9%, Solvay), poly(ethylene glycol) dimethyl ether (PEGDME, 500 g mol^−1^, Sigma‐Aldrich), allyl boronic acid pinacol ester (AAPE, TCI), poly(ethylene glycol) methyl ether methacrylate (PEGMEMA, 500 g mol^−1^, Sigma‐Aldrich), and PSS‐octavinyl substituted (POSS, MACKLIN) were used as received. Lithium metal and aluminum foil (battery grade) were supplied by UBIQ.

### 2.2 Deposition of QSPEs and CPEs onto the Lithium Metal Anode

**Scheme 1:**
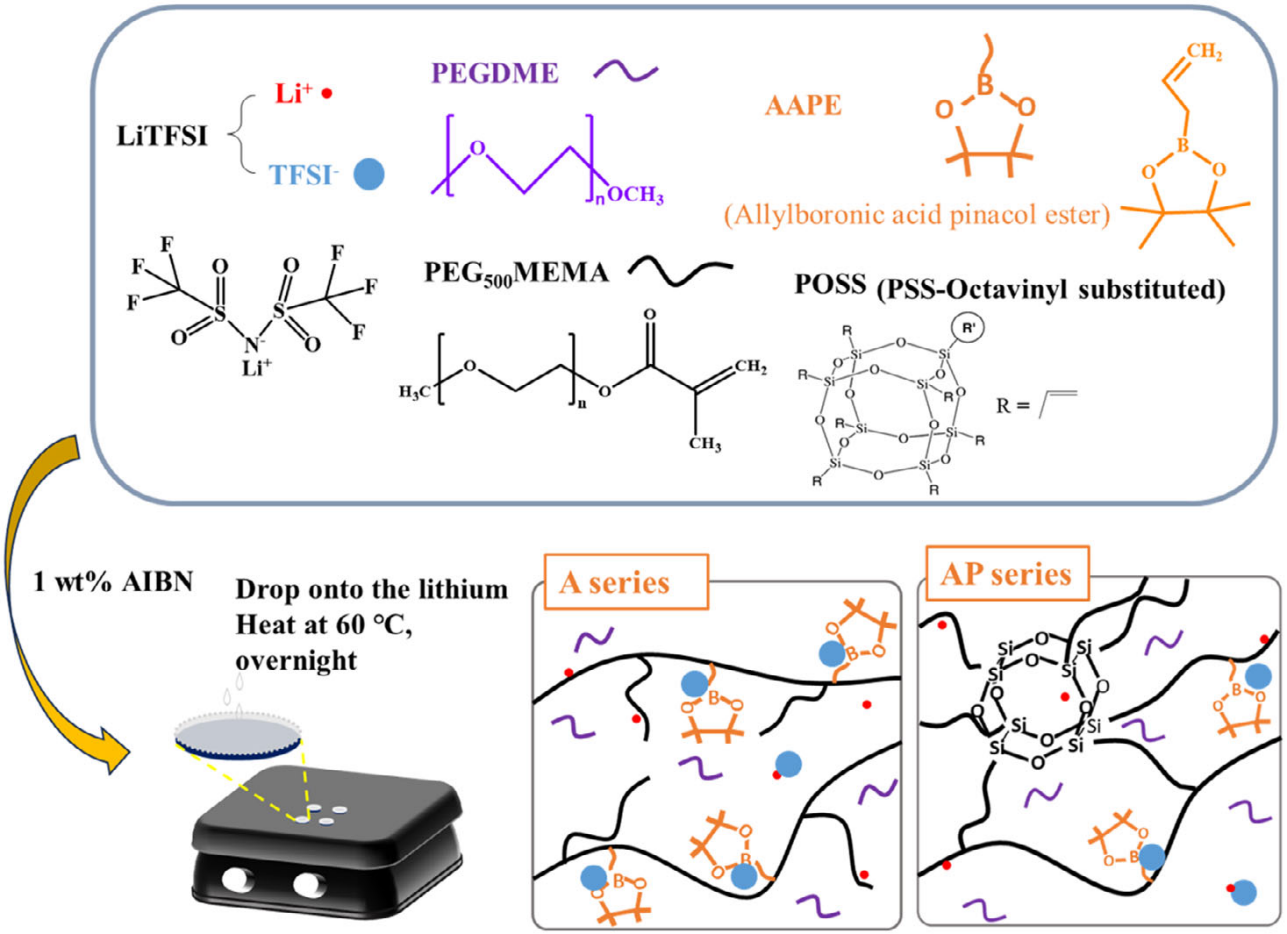
Chemical structures of monomers, plasticizer, and initiator and schematic illustration of the preparation of QSPEs (A series) and CPEs (AP series) onto the lithium metal.

As shown in **Scheme** **1** and Table S1, Supporting Information, the precursor solutions were prepared by mixing various mass ratios of PEGMEMA, AAPE, and POSS as the monomers, PEGDME as the plasticizer, LiTFSI as the lithium salt, and AIBN (1 wt% of the sum of monomers) as the thermal initiator. The QSPEs and CPEs were fabricated via in situ thermal polymerization method, which is a well‐known free radical vinyl polymerization method.^[^ ^37^ ^]^ The as‐prepared precursor solution (50 μL) was directly dropped on the lithium metal, followed by heating at 60 °C in an oven overnight (Scheme 1). The QSPE‐ or CPE‐coated lithium metal samples were obtained after thermal polymerization.

### 2.3 Preparation of Electrode and Cell Assembly

For the preparation of the slurry, the ratio between the active material (LFP), conductive carbon (super P), and binder (PVDF) was 8:1:1 with the NMP as the solvent. The binder, super P, and LFP were sequentially added to the solvent and the resultant mixture was stirred at 2000 rpm for 30 min to prepare the slurry. Then the slurry was poured onto an aluminum foil and coated using an automatic coater with a coating speed of 10 mm s^−1^ and a 105 μm‐thick blade. The coated foil was placed in a vacuum oven at 100 °C overnight to remove the solvent. The electrode was compacted by a wheel roller with the gap fixed at 40 μm. Finally, the electrode was cut into a circular shape with 13 mm in diameter with the mass loading of LFP approximately between 2.0 and 2.3 mg cm^−2^. The CR2032 coin cells were assembled in an argon filled glove box by stacking the LFP cathode and QSPE‐coated (or CPE‐coated) lithium metal anode in order.

### 2.4 Characterization

Fourier transform infrared spectrometry (FT‐IR, thermo‐Nicolet Nexus 5700) was used to measure the IR spectra at the wavenumber ranging from 650 to 3500 cm^−1^. X‐ray diffractometer (XRD, Rigaku Ultima IV‐9407F701) was used to characterize the crystallinity of lithium salt in PEs with Cu Kα radiation (0.154 nm), operating at 40 kV and 30 mA at the scan rate of 4° per minute from 5° to 80°. Compression testing was done by a dynamic mechanical analyzer (DMA, TA‐RSA‐G2) to observe the mechanical properties of the electrolyte membranes with a compression rate of 0.01 mm s^−1^. The cylindrical‐shaped samples were prepared with 10 mm of diameter and 5 mm of thickness. The measurements of membrane thermal properties were carried out by a thermogravimetric analysis instrument (TGA, Perkin‐Elmer TGA 4000) to record the thermal stability of the membranes. The samples were heated to 600 °C with a heating rate of 10 °C min^−1^ under nitrogen atmosphere. Scanning electron microscope (SEM, Hitachi SU‐8010) was used to observe the surface morphology of electrolyte membrane and lithium metal before and after cycling test. The glass transition temperatures (*T*_g_) of the QSPE and CPE samples were measured by a low‐temperature differential scanning calorimeter (LT‐DSC, Netzsch 204 F1) with the temperature heated from −100 to 50 °C.

### 2.5 Electrochemical Measurements

The battery test was carried out by using a battery testing system (BAT‐750B) with the voltage from 2.5 to 4 V, and the charge/discharge tests were performed at various C‐rates from 0.1 to 5 C and the cycling tests were performed at 0.2 C. The linear sweep voltammetry (LSV) analysis was used to determine the electrochemical stability window, and the coin cells were assembled without electrode to avoid the additional influence and tested by using an AC impedance spectroscopy (SP‐300, Bio‐Logic) at the scan rate of 5 mV s^−1^ from 0 V versus Li/Li^+^ to 6 V versus Li/Li^+^. To calculate the ionic conductivity, the resistance was measured by using a SP‐300 AC impedance spectroscopy. From open‐circuit‐voltage, the amplitude was set at 5 mV, and the frequency range was from 0.1 Hz to 1 MHz at the temperature from 30 to 80 °C. The electrolyte membrane was sandwiched between two stainless steel plates for the measurements. After the measurement of the resistance (*R*), the ionic conductivity (*σ*) was calculated by Equation (1), where *L* is the thickness of the electrolyte membrane and *A* is the contact area between the electrolyte and electrode.

(1) $$\sigma =\frac{L}{R\times A}$$

Electrochemical impendence spectroscopy (EIS) of Li|QSPE|LFP or Li|CPE|LFP cells were measured by using a SP‐300 AC impedance spectroscopy with the amplitude of 5 mV and the frequency from 0.1 to 10 MHz at room temperature. The lithium transference number measurements of the symmetrical lithium cells were conducted by using a SP‐300 AC impedance spectroscopy. The lithium transference number ($T_{{\text{Li}}^{+}}$) were calculated by Equation (2), where *I*_0_ and *I*_s_ are current before and after polarization, *R*_0_ and *R*_s_ are resistance values before and after polarization, and Δ*V* is the applied voltage (0.01 V).

(2) $$T_{{\text{Li}}^{+}}=\frac{I_{s}\left(\Delta V-I_{0}R_{0}\right)}{I_{0}\left(\Delta V-I_{s}R_{s}\right)}$$

## 3 Results and Discussion

### 3.1 Characterization of QSPEs

**Figure 1:**
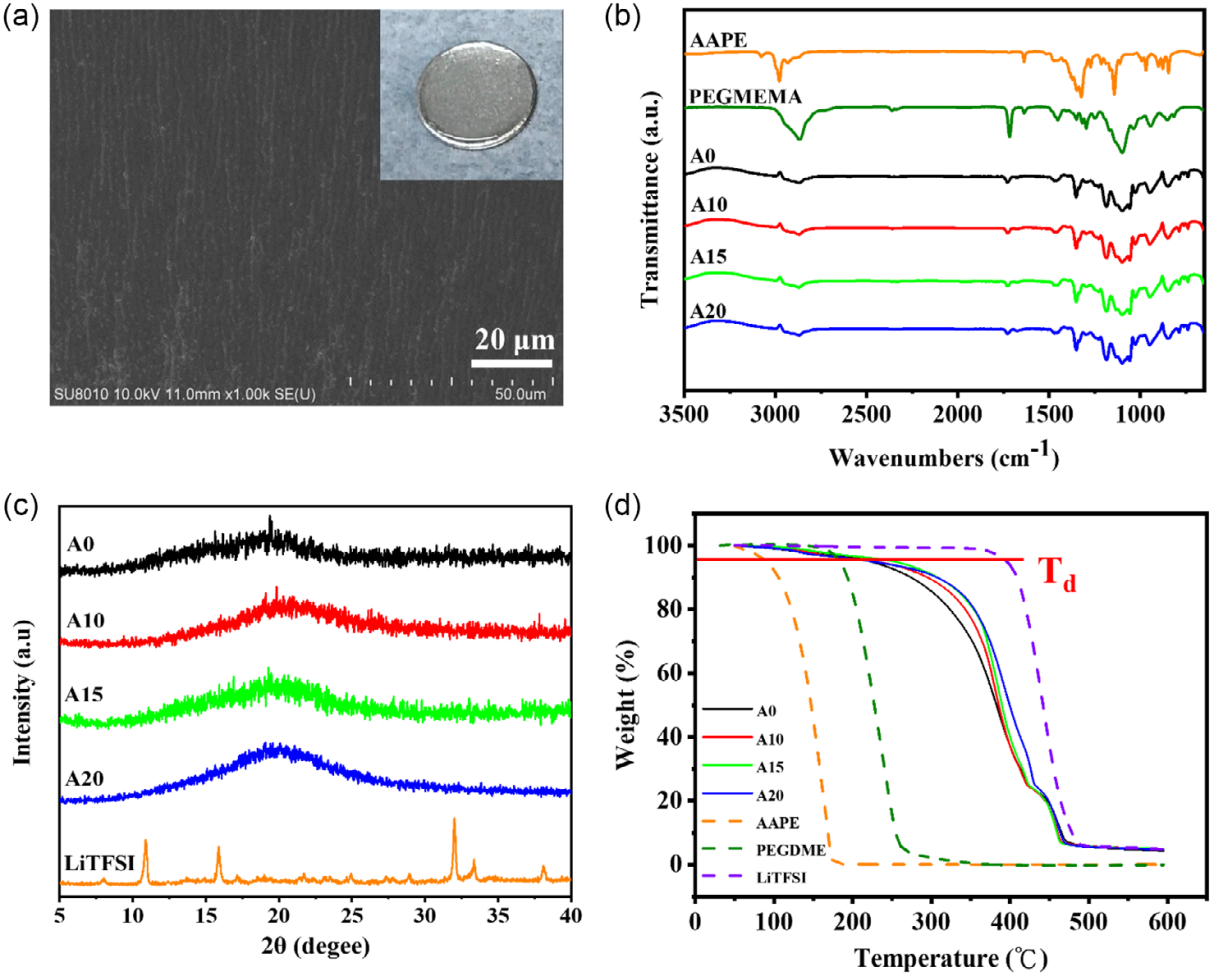
a) SEM and optical images of A10 QSPE membrane coated onto Li anode. b) FT‐IR spectra of A‐series membranes and monomers between 650 and 3500 cm^−1^. c) XRD patterns of A‐series membranes and lithium salt. d) TGA curves of A‐series membranes, monomers, and lithium salt.

The A‐series QSPE membranes were prepared by depositing the precursor solution directly on the lithium anode, followed by thermal polymerization overnight. The optical image showed that the QSPEs were conformally deposited onto the lithium anode and the SEM image revealed the smooth surface of the membrane (**Figure** **1**a). The analysis of FT‐IR was conducted to ensure whether the in situ polymerization was completed at 60 °C overnight. In Figure 1b, the A‐series membranes exhibited the absorption of –(CH_2_)– groups at 2700–3000 cm^−1^ and the absorption of BO, bending of B—C, and C—O bonds at around 1324 cm^−1^. The C=O stretching peak in PEGMEMA monomer was at 1718 cm^−1^, and the signal at around 1640 cm^−1^ in AAPE and PEGMEMA monomers corresponded to the absorption of C=C stretching. From the FT‐IR spectra, the C=C stretching peaks in both monomers disappeared after the thermal polymerization, indicating that the reaction was completed under the condition of 60 °C overnight. XRD analysis of the QSPEs showed the presence of a broad diﬀraction peak ranging between at 2*θ *= 17–22° and the disappearance of the diﬀraction peaks corresponding to LiTFSI, revealing that all the membranes were in an amorphous state (Figure 1c). The results indicated that LiTFSI salt was miscible with the polymer matrix upon thermal polymerization, consistent with the SEM analysis showing that the membranes exhibited smooth surface (Figure 1a). The TGA profiles of the QSPEs showed a two‐stage weight loss at 180–400 °C and above 400 °C (Figure 1d). The initial weight was attributed to the decomposition of the added PEGDME segments in the polymer matrix and the polymerized AAPE and PEGMEMA moieties, while the secondary weight loss originated from the decomposition of LiTFSI above 400 °C. Their decomposition temperatures at 5% weight loss, denoted as *T*_d_, were ranged between 220 and 230 °C, which were much higher than those of additive and monomers. This is mainly attributed to the entrapment of PEGDME additive in the intertwined polymer matrix and the tether of the monomers onto the networked polymer chains.^[^ ^26^, ^38^ ^]^ Moreover, the additive might exhibit interactions with the constituents in the QSPEs, leading to the slower decomposition. DMA measurements showed that the addition of borate monomer led to the decrease in the mechanical properties and all the QSPEs exhibited excellent elasticity, evidenced by the resistance to deformation upon the loading and unloading (Figure S1, Supporting Information). DSC analysis was conducted to determine the *T*_g_ values of all QSPE samples and the data were summarized in Table S2, Supporting Information. The A10 sample (−61.2 °C) exhibited lower *T*_g_ value than the A0 counterpart (−56.5 °C) (Table S2 and Figure S2, Supporting Information), suggesting that the addition of 10 wt% AAPE possibly resulted in a loose polymer skeleton and the increase in the amorphous fraction of the copolymer.^[^ ^21^, ^29^ ^]^ This would be beneficial for ion transport. However, the *T*_g_ value increased with the increment in the weight percentage of AAPE (Figure S2, Supporting Information), indicating that the increase in AAPE could lead to a denser network in the polymer skeleton.

### 3.2 Electrochemical Performances of QSPE

**Figure 2:**
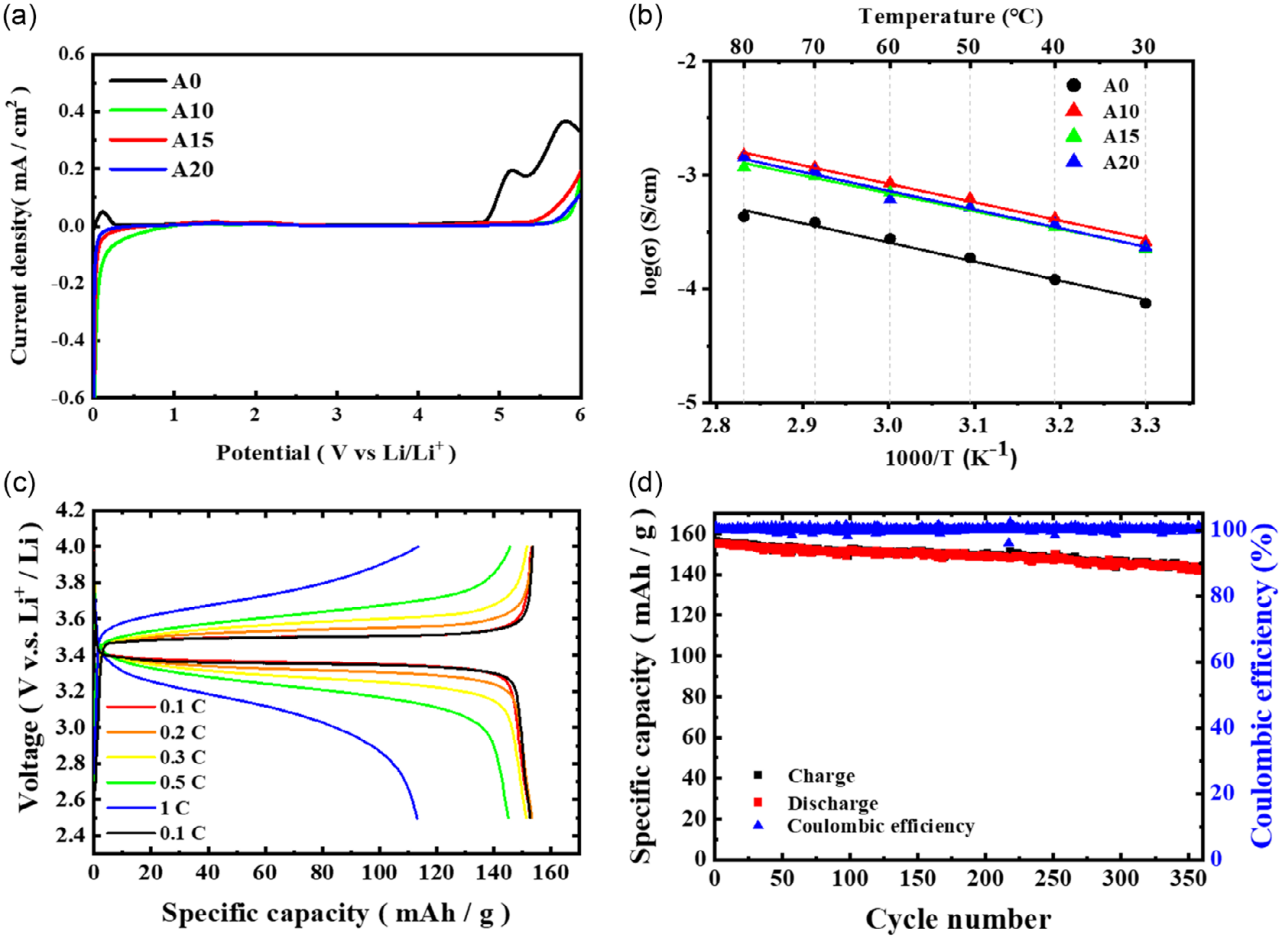
a) The LSV curves of A‐series QSPEs in the range of 0–6 V. b) Temperature dependence of ionic conductivity between 30 and 80 °C for A‐series QSPEs; c) Charge and discharge curves and d) cycling performance of Li|A10|LFP cell at 25 °C.

The stability of the A‐series membranes in the range of the potential from 0 to 6.0 V (vs Li/Li^+^) was analyzed by LSV measurements. As shown in **Figure** **2**a, the A10, A15, and A20 QSPEs exhibited the upper limit of the electrochemical voltage window to 5.3 V (vs Li/Li^+^), whereas the A0 counterpart exhibited upper limit of the voltage window to 4.6 V (vs Li/Li^+^). The results showed that the incorporation of borate monomer led to the extension of electrochemical voltage window. Previous studies have also found that the addition of borate monomer could enhance the electrochemical stability window of the compound.^[^ ^21^, ^22^ ^]^ The ionic conductivities of A‐series membranes were measured at the temperature ranging between 30 and 80 °C as shown in Figure 2b. It was found that the incorporation of boron moiety resulted in the increase in ionic conductivity and the A10 membrane exhibited the highest ionic conductivity among all the samples. The results were consistent with the trend in *T*_g_ value, showing that the A10 membrane exhibited the lowest *T*_g_ value. The logarithm to the values of the ionic conductivities was found to exhibit a linear relationship with the reciprocal of temperature (1000/(*T*–*T*_g_)), suggesting that the data conformed to the VTF (Vogel‐Tammann‐Fulcher) equation.^[^ ^39^ ^]^ Analysis of the fitted plots revealed that the ionic conductivity increased with the increment of the temperature, which could increase the free volume within the system and promote the segmental motion of polymer segments, making it easier for ions to hopping from one position to another between and within segments.^[^ ^40^ ^]^ Moreover, the anions could be immobilized by boron with an empty p‐orbital via electronic interaction,^[^ ^31^, ^41^ ^]^ which would facilitate the movement of Li cations.

The lithium‐ion transference number (*t*_Li_^+^) plays a central role in assessing the lithium‐ion conduction capability of an electrolyte. During the charge and discharge of the battery, the movement of lithium ions must overcome various resistances, such as interface impedance between the electrode and electrolyte, as well as transmission impedance within the electrolyte. A higher number of lithium‐ion migrations represents a faster ion movement speed. Figure S4, Supporting Information, showed the polarization current‐time and the corresponding electrochemical impedance spectra before and after polarization of Li|Ax|Li cells, and the relevant data are summarized in **Table** **1**. The lithium‐ion transference numbers for A10, A15, and A20 were calculated to be 0.321, 0.355 and 0.346, respectively, which were much higher than that for A0 (0.27). The results indicated a significant enhancement in the lithium‐ion transference number upon the incorporation of boron moiety. It is worth noting that the both A15 and A20 membranes exhibited comparable lithium‐ion transference number, suggesting the further increasing of the boron moiety did not promote the lithium‐ion migration. The primary contribution to facilitating lithium‐ion transportation is the Lewis acidic properties of the boron moiety, which enable it to capture anions. This assists in the dissociation of lithium salts, leading to an enhancement in the rate of lithium‐ion transportation.^[^ ^31^, ^41^ ^]^

**Table 1:** Lithium‐Ion transference number and initial discharge capacity of QSPEs and CPEs.

Sample	Lithium‐ion transference number					Initial Discharge capacity [mAh g^−1^] at 25 °C	
	*I*_0_ [mA]	*I*_s_ [mA]	*R*_0_ [Ω]	*R*_s_ [Ω]	$T_{{\text{Li}}^{+}}$	0.1 C	0.5 C
A0	0.26	0.09	10.8	11.2	0.270	153.4	124.1
A10	0.26	0.11	13.28	13.87	0.321	152.7	145.1
A15	0.35	0.17	12.11	11.80	0.355	152.1	146.3
A20	0.34	0.18	15.48	13.79	0.346	153.2	138.7
A10P1	0.20	0.11	26.26	22.51	0.316	150.6	143.5
A10P3	0.25	0.14	23.94	20.89	0.312	139.1	134.7
A10P5	0.28	0.11	15.35	14.30	0.268	125.4	109.9

### 3.3 Battery Performances of Li|QSPE|LFP Cells

The electrochemical performances of the Li|QSPE|LFP cells were assessed by presenting the charge‐discharge curves of the Li|QSPE|LFP cells. After activating at a current rate of 0.1C for two cycles to establish a stable SEI layer, the Li|QSPE|LFP cells were charged and discharged at different C‐rates with the operating voltage set at 2.5–4.0 V and at 25 °C. As shown in Figure 2c, S5a,b, and S6a, Supporting Information, the discharge capacities of Li|A10|LFP, Li|A15|LFP, and Li|A20|LFP cells were comparable with that of Li|A0|LFP counterpart at 0.1 C (Table 1), whereas at the C‐rates higher than 0.1 C their discharge capacities were higher than that of Li|A0|LFP counterpart except the Li|A20|LFP at 1 C (20 mAh g^−1^). The Li|A10|LFP and Li|A15|LFP cells were able to maintain a discharge capacity slightly higher than 110 mAh g^−1^ at 1 C (Figure 2c and S5a, Supporting Information). The long‐term cyclic tests at 0.2 C were conducted to assess the duration of battery life. During repeated charge‐discharge processes, lithium ions continuously migrate in and out. The ionic conductivity of the electrolyte and the uniformity of the SEI layer are critical factors affecting their electrochemical performance. The Li|A10|LFP, Li|A15|LFP, and Li|A20|LFP cells exhibited more stable discharge capacities than Li|A0|LFP counterpart and nearly 100% coulombic efficiency (Figure 2d, S5c,d, and S6b, Supporting Information). In contrast, the cycling performance of the Li|A0|LFP cell showed a continuous decline in discharge capacity after repeated charge‐discharge cycles and a significant decrease in coulombic efficiency after 70 cycles (Figure S6b, Supporting Information). The results may possibly due to the truth that the anion immobilization and in situ thermal polymerization might exert synergistic effects on decreasing the lithium ion concentration gradient from the bulk electrolyte to the anode surface, which could reduce the electric field at the metal electrode and further lead to uniform Li deposition behavior.^[^ ^31^, ^42^, ^43^ ^]^ Among all samples, the Li|A 10|LFP cell exhibited the best cycling performance, evidenced by the highest capacity retention rate of 95% and 92% at 250 and 350 cycles, respectively (Figure 2d). It can be attributed that the A10 membrane exhibited the highest ionic conductivity among all the samples. The electrochemical impedances of the A10 membranes via both in situ and ex situ methods were measured during the cycling test at 0.2 C for every 20 cycles (Figure S7, Supporting Information). The impedance curves exhibited a combination of an arc and a sloped line, where the arc was composed of two semicircles of PE interface impedance (*R*_SEI_) and charge transfer impedance (*R*_ct_). It could be observed that the A10 membrane prepared via ex situ method exhibited higher impedance than that prepared via in situ method. The results highlight that the PEs prepared via in situ method exhibit improved interfacial contact and electrochemical performance.

**Figure 3:**
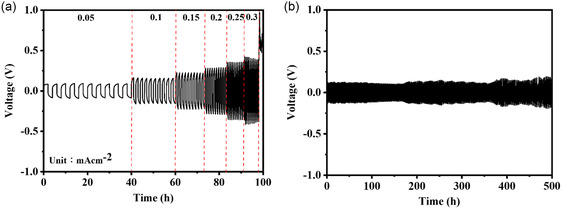
a) Lithium stripping/plating curves of symmetric Li|A10|Li cell at diﬀerent current densities. b) Cycle performance graph of symmetric Li|A10|Li cell at 0.1 mA cm^−2^.

The symmetric Li|A10|Li cell was chosen to conduct the plating and stripping test and the results were presented in **Figure** **3**. The constant current density charge‐discharge cycle test showed that the overvoltage increased as the current rate increased, and the electrolyte could tolerate until the current rate of 0.3 mA cm^−2^ (Figure 3a). The detected overvoltage values at the current density of 0.1 and 0.2 mA cm^−2^ were around 130 and 250 mV, respectively. The cyclic charge‐discharge tests were conducted on the cell at 25 °C using a fixed current density of 0.1 mA cm^−2^. The relationship between voltage and time was monitored to assess the endurance of the symmetric cell at the magnitude of overvoltage. The symmetric Li|A10|Li cell exhibited stable charge and discharge behavior for 500 h (Figure 3b), showing the good stability and durability of the system.

### 3.4 Electrochemical Performances of CPEs

**Figure 4:**
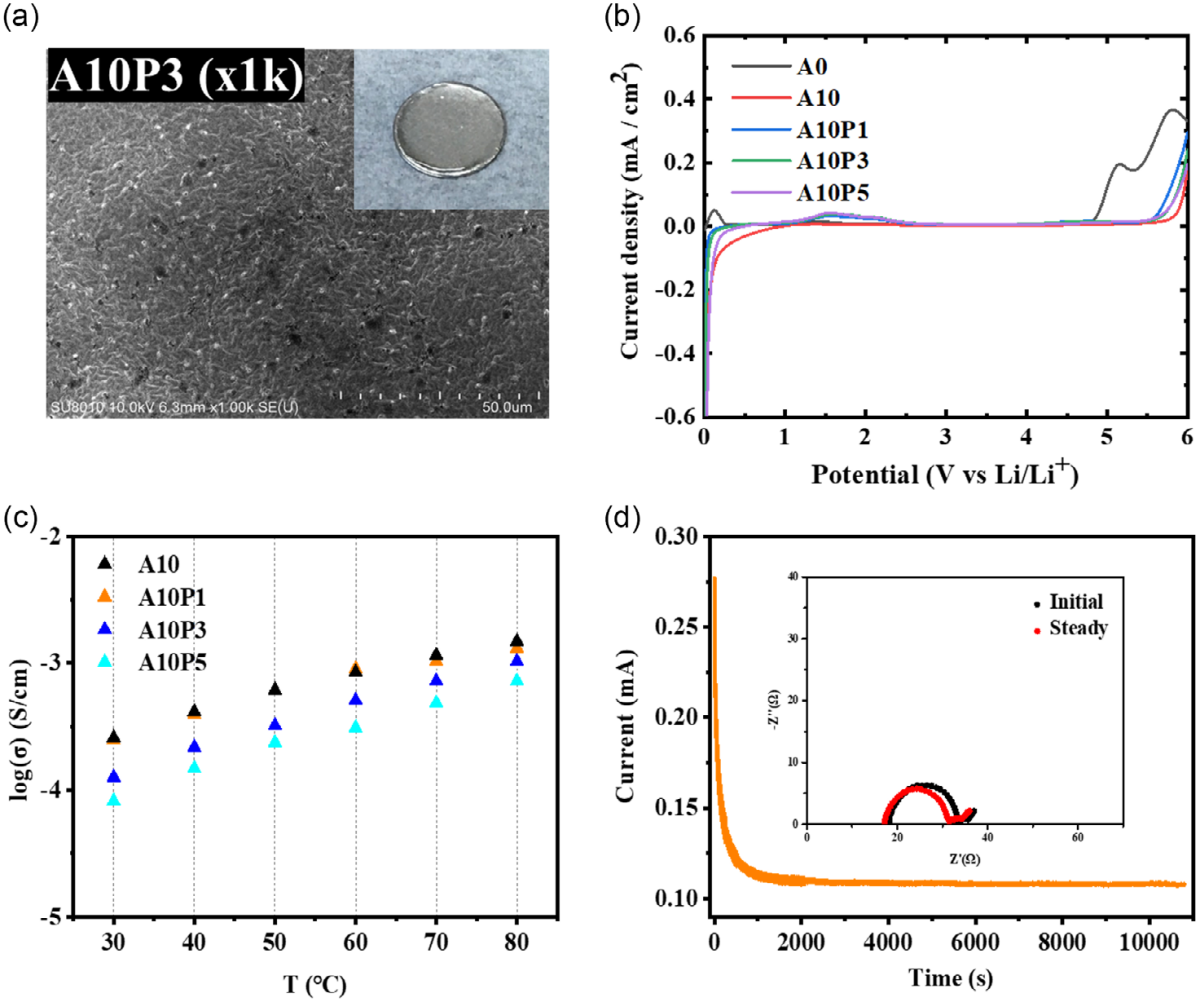
a) SEM and optical images of A10P3 CPE membrane coated onto Li anode. b) The LSV curves of AP‐series CPE membranes in the range of 0–6 V. c) Temperature dependence of ionic conductivity between 30 and 80 °C for AP‐series CPEs. d) Chronoamperometry profile of A10P3 CPE membrane.

The above results show that the A10 QSPE exhibited a stable long‐term charge‐discharge performance. As shown in Scheme 1, POSS with eight allyl groups was then incorporated into the A10 QSPE in an attempt to further improving the electrochemical performance. The A10P1, A10P3, and A10P5 (AP‐series) membranes were prepared by adding additional 1%, 3%, and 5% of POSS monomer with respect to the total of the AAPE and PEGMEMA monomers, where the detailed proportions were listed in Table S1, Supporting Information. The precursor solution was directly deposited onto the lithium anode, followed by the in situ thermal copolymerization to form the CPE‐coated lithium anode. The CPEs were conformally deposited onto the lithium anode and the CPE membranes exhibited smooth surfaces based on SEM analysis (for example, **Figure** **4**a). The TGA analysis revealed that the CPEs exhibited comparable thermal stability with A10, as evidenced by the comparable percentages of weight loss for the CPEs with that of A10 (Figure S8, Supporting Information). The results suggested that a small amount of added POSS did not improve their thermal stability. The LSV measurements of the AP‐series membranes were conducted in the range of the potential from 0 to 6.0 V (vs Li/Li+). As shown in Figure 4b, the upper limit of the electrochemical voltage window for all the AP‐series CPEs could be up to 5.4 V (vs Li/Li^+^), which is comparable with that of the A10 one. The ionic conductivities of AP‐series membranes showed that the ionic conductivity slightly decreased with the increase in POSS content (Figure 4c). The copolymerization of POSS with AAPE and PEGMEMA monomers might result in the decrease in the segmental movement, which would retard the movement of Li cations. DSC analysis was conducted to determine the *T*_g_ values of all CPE samples. It was found that the *T*_g_ value increased with the increment in the amount of added POSS (Table S2 and Figure S3, Supporting Information), indicating the addition of POSS could lead to a denser network in the polymer skeleton due to the slight increase in the crosslinking density. Therefore, the higher concentrations of POSS could possibly lead to higher crosslinking density or interface effects, thereby restricting lithium‐ion migration. The logarithm of the values of the ionic conductivities was found to exhibit a linear relationship with the reciprocal of temperature (1000/(*T–T*_g_)) (Figure S9, Supporting Information), suggesting that the data conformed to the VTF (Vogel‐Tammann‐Fulcher) equation.^[^ ^39^ ^]^ The addition of small amount of POSS did not alter the mechanism of Li transport in the CPEs.

The lithium‐ion migration numbers of the AP‐series membranes were analyzed to assess whether the addition of POSS improves the lithium‐ion migration performance (Table 1, Figure 4d and S10, Supporting Information). The lithium‐ion transference numbers of A10P1 and A10P3 were determined to be 0.316 and 0.312, respectively, which were slightly lower than that of A10 (0.321). The addition of small among of POSS did not perturb the lithium‐ion migration performance in the CPEs. However, the lithium‐ion transference number drastically decreased to 0.268 upon the addition of 5 wt% POSS. One possible reason for this could be the high crosslinking density, which may affect their segmental movement, dispersion, and interaction in the electrolyte, thereby hampering the lithium‐ion migration performance.

### 3.5 Battery Performances of Li|CPE|LFP Cells

**Figure 5:**
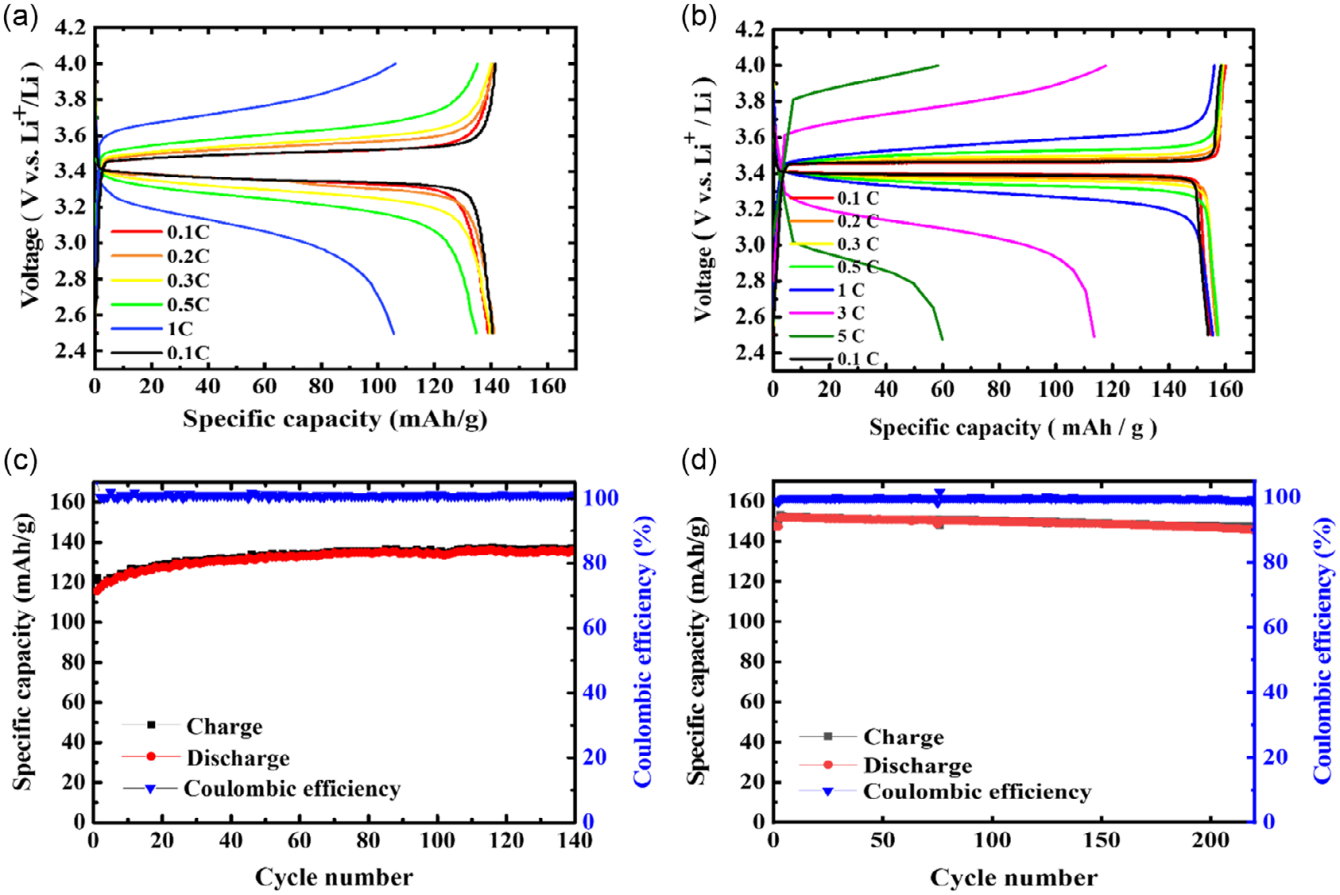
a,b) Charge and discharge curves and c,d) cycling performance of Li|A10P3|LFP cell at (a,c) 25 and (b,d) 60 °C.

The electrochemical performances of the Li|CPE|LFP cells were assessed by presenting the charge‐discharge curves of the Li|CPE|LFP cells at different C‐rates with the operating voltage set at 2.5–4.0 V and the temperature at 25 °C. As shown in Table 1 and Figure S11a, Supporting Information, the discharge capacities of Li|A10P1|LFP cell were comparable with those of A10 counterpart at low C‐rates (0.1–0.3 C), whereas the discharge capacities at the C‐rates higher than 0.5 C were higher than those of A10 counterpart. With the addition of 1 wt% POSS, the presence of oxygen atoms in POSS structure could facilitate lithium‐ion conduction and the polyhedral structure of POSS could establish continuous ion pathways,^[^ ^31^, ^32^ ^]^ resulting in the enhanced electrochemical performances of A10P1. However, upon increasing the POSS weight percentage (3 and 5 wt%), the discharge capacities of the Li|CPE|LFP cells decreased regardless of the C‐rate (**Figure** **5**a and S12a, Supporting Information), The ionic conductivity and lithium‐ion transference number also exhibited the same trend. At 25 °C, higher concentrations of POSS could possibly lead to higher crosslinking density and interface effects, resulting in the decrease in the lithium‐ion migration performance. The long‐term cycling tests of the Li|CPE|LFP cells were conducted at a fixed rate of 0.2C and at 25 °C. The results revealed that all systems achieved coulombic efficiency of nearly 100% (Figure 5c, S11c, and S12c, Supporting Information). It was observed that the Li|CPE|LFP cells exhibited lower discharge capacities in the initial stage and gradually became stable at fixed discharge capacities after a certain period of cycling. The Li|A10P1|LFP cell achieved a long cycle life (180 cycles) with a capacity of 143.2 mAh g^−1^ at 0.2 C.

**Figure 6:**
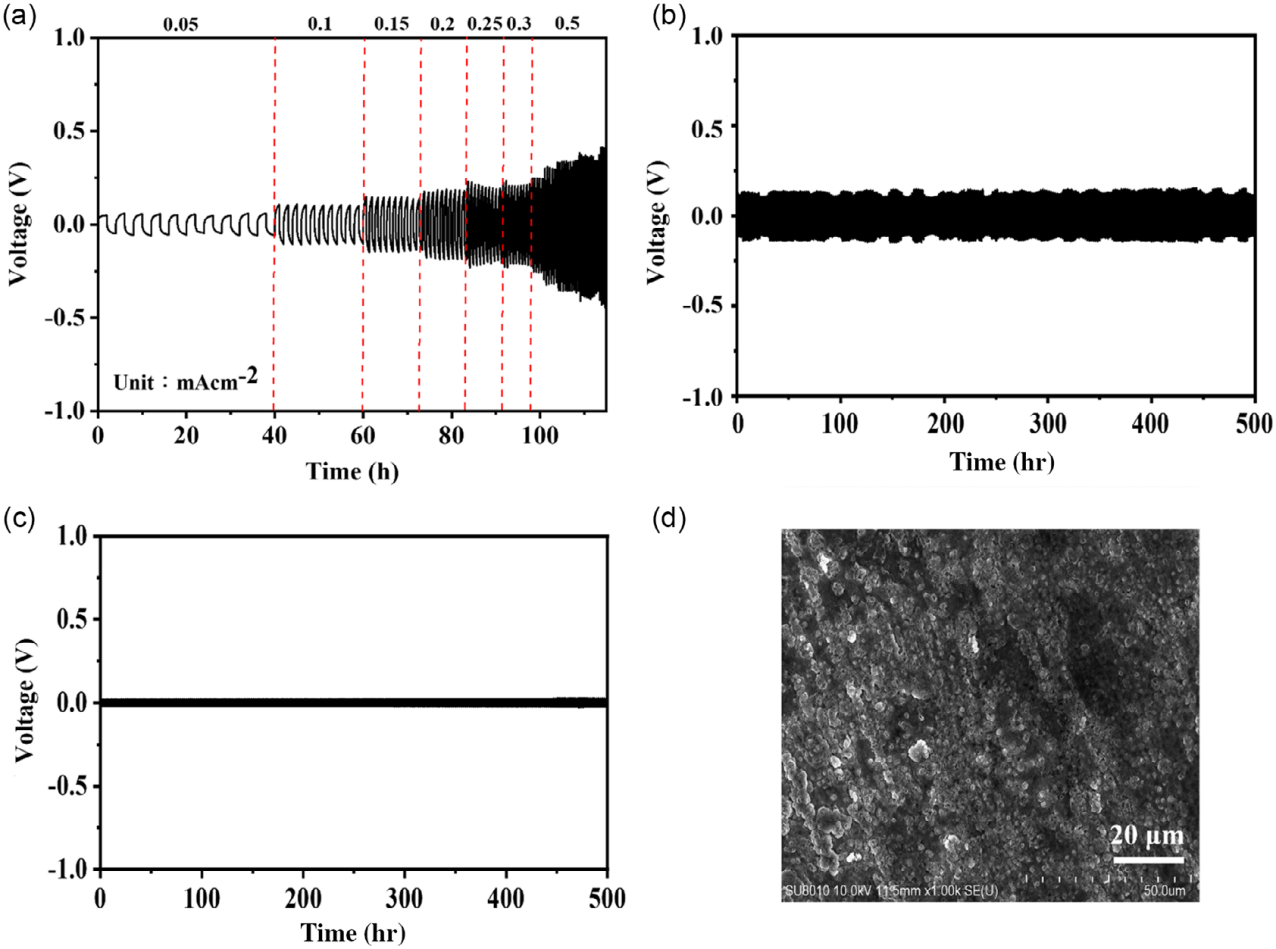
a) Lithium stripping/plating curves of symmetric Li|A10P3|Li cell at diﬀerent current densities under 25 °C. b) Cycle performance graph of symmetric Li|A10P3|Li cell at 0.1 mAcm^−2^ under (b) 25 and c) 60 °C. d) SEM image of the lithium anode for the symmetric Li|A10P3|Li cell after 100 h cycle test at 0.1 mA cm^−2^ under 25 °C.

The electrochemical performances of the Li|CPE|LFP cells were also assessed at an elevated temperature (60 °C). As shown in Figure 5b, S11b, and S12b, Supporting Information, the increase in temperature resulted in the increase in the discharge capacity for all the Li|CPE|LFP cells and the cells could be operated at higher C‐rates (3 and 5 C). The Li|A10P1|LFP and Li|A10P3|LFP cells exhibited higher discharge capacities than the Li|A10|LFP counterpart at a given C‐rate (**Figure** **6**b, S11b, and S13a, Supporting Information). The incorporation of optimal POSS and increase in temperature could synergistically facilitate the segmental movement, lithium‐ion conduction and the establishment of continuous ion pathways,^[^ ^31^, ^32^ ^]^ leading to the enhanced electrochemical performance at 60 °C. The long‐term cycling tests of the Li|CPE|LFP cells were conducted at a fixed rate of 0.5 C. The results showed that all systems achieved coulombic efficiency of nearly 100% and the Li|A10P3|LFP cell exhibited the best electrochemical performance among all the Li|CPE|LFP cells (Figure 5d, S11d, and S12d, Supporting Information). The Li|A10P3|LFP cell exhibited stable discharge capacity within 220 cycles (Figure 5d), whereas the discharge capacities of Li|A10P1|LFP and Li|A10P5|LFP cells gradually decreased after 50 cycles. For comparison, the long‐term cycling test of the Li|A10|LFP cell was conducted at the same condition and the results showed that the Li|A10|LFP cell experienced a sudden drop in discharge capacity at 135 cycles (Figure S13b, Supporting Information). The A10P3 CPE with optimal POSS content might be able to maintain the structural stability in long‐term use within batteries at the elevated temperatures.

The symmetric Li|A10P3|Li cell was chosen to conduct the plating and stripping test at 25 °C. As shown in Figure 6a, the constant current density charge‐discharge cycle test showed that the overvoltage raised as the current rate increased, and the CPE could tolerate until the current density of 0.5 mA cm^−2^, which was higher than the Li|A10|Li counterpart. At a fixed current density, the detected overvoltage value for the symmetric Li|A10P3|Li cell was lower than that for Li|A10|Li counterpart (Figure 3a and 6a). The cyclic charge‐discharge tests were conducted on the cell at 25 and 60 °C using a fixed current density of 0.1 mA cm^−2^. As shown in Figure 6b,c, the symmetric Li|A10P3|Li cell exhibited stable charge and discharge behavior for 500 h, showing the good stability and durability of the system. It is worth noting that the detected overvoltage value at 60 °C was about 20 mV, which is much lower than that at 25 °C. After a 100 h lithium dendrite test, the Li|A10P3|Li cell was disassembled to extract the lithium metal layer. SEM analysis showed that the lithium metal deposition exhibited a uniform surface as compared to the fresh lithium anode (Figure 6d and S14, Supporting Information), hence possessing superior long‐term performance. The observed improvements could be attributed to several factors. Firstly, the addition of POSS promoted the formation of a stable and protective SEI layer which played a critical role in preventing electrolyte decomposition and excessive lithium deposition on the electrode surface, thereby reducing overvoltage and mitigating battery capacity decay. Secondly, the oxygen atoms presented in the structure of POSS could form bonds with lithium ions, enhancing lithium‐ion conductivity within the CPEs.

**Figure 7:**
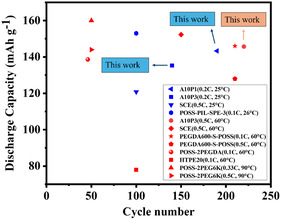
Comparisons of the discharge capacity and cycle life of the Li|LFP cells based on A10P1 and A10P3 CPEs with those of others reported in the literature at 25 °C (blue symbols) and 60 °C (red symbols). The detailed information about the above materials was summarized in Table S3, Supporting Information.

**Figure** **7** and Table S3, Supporting Information show the comparison of the performance of Li|LFP cells based on AP‐series CPEs with recently reported CPEs based on POSS.^[^ ^12^, ^21^, ^44^, ^45^, ^46^, ^47^ ^]^For the cells operating at 25 °C, the A10P1 CPE endowed the equipped cells achieving the longest cycle life (180 cycles) without capacity loss among all the reported studies. It is worth noting that the Li|A10P3|LFP one also exhibited a stable cycle life within 140 cycles without capacity loss. For those operating at 60 °C, the A10P3 CPE endowed the equipped cells achieving the longest cycle life (220 cycles) with a high capacity (145.7 mAh g^−1^) and almost no capacity loss (99.0%) among all the reported studies. It can be seen that the Li|PEGDA_600_‐S‐POSS|LFP cell could achieve a cycle life (210 cycles) with a capacity retention of 95%.^[^ ^41^ ^]^ However, the capacities of Li|PEGDA_600_‐S‐POSS|LFP at 0.1 and 0.5C were 146 and 128 mAh g^−1^, respectively,^[^ ^41^ ^]^ which were much lower than those of Li|A10P3|LFP one. The cell based on AP‐series CPEs with optimal POSS content exhibited the combination of a high capacity and a long cycle life, making it a breakthrough for improving current CPE systems.

## 4 Conclusions

This study demonstrated that the QSPEs containing boron moiety exhibit higher electrochemical voltage window, ionic conductivity, and transference number as well as better electrochemical performance than the PEG‐based counterpart due to the synergistic effect between the incorporation of anion‐trapping boron moiety and in situ polymerization. The Li|A10|LFP cell achieved 350 cycles of long‐term charge‐discharge at 0.2 C under 25 °C, maintaining a high capacity retention of 91.9%, and exhibited stable lithium plating‐stripping over 500 h. The primary mechanisms through which the QSEs facilitated lithium‐ion conduction were in the following. Due to the Lewis acid effect, the boron moiety could capture anions and promote the dissociation of lithium salts, allowing more lithium ions to be in the free state, thereby enhancing the lithium‐ion conductivity. The polyether segments within PEG could facilitate lithium‐ion transfer through a hopping mechanism generated by segmental motion. The CPEs were then prepared based on the composition of A10 QSPE with added POSS. POSS containing oxygen atoms in its structure exhibited excellent thermal stability, and its polyhedral structure contributed to the formation of continuous ion channels, providing pathways for lithium‐ion transport. It was found that the CPEs with an optimal addition of POSS exhibited lower overpotential during the lithium plating‐stripping test and better electrochemical performance than the QSPE counterparts. The Li|A10P3|LFP cell exhibited the best electrochemical performance among the Li|CPE|LFP cells with stable discharge capacity of up to 220 cycles at 60 °C. The optimal POSS addition could facilitate the lithium‐ion conduction and establishment of continuous ion pathways, further improving their electrochemical performance. Further increasing the POSS amount would increase the crosslinking density, creating obstacles to lithium‐ion conduction and thus reducing the electrochemical performance. Future research could further explore the application of POSS in other battery systems and optimize the proportion and structural design of POSS to enhance battery performance.

## Conflict of Interest

The authors declare no conflict of interest.

## Author Contributions

**Chia‐Chi Chang**: Conceptualization (supporting); Formal analysis (Lead); Investigation (equal); Writing—original draft (equal). **Min‐Hsien Shen**: Data curation (lead); Formal analysis (equal); Investigation (equal); Writing—original draft (equal). **Yuan‐Shuo Hsu**: Formal analysis (supporting); Investigation (supporting); Validation (equal). **Hsisheng Teng**: Funding acquisition (supporting); Validation (supporting); Writing—review and editing (supporting). **Jeng‐Shiung Jan**: Conceptualization (lead); Funding acquisition (lead); Project administration (Lead); Supervision (lead); Writing—review and editing (lead).

## Supporting information

Supplementary Material

## Data Availability

The data that support the findings of this study are available from the corresponding author upon reasonable request.
