# Supplementary material for: In Situ Formed Composite Polymer Electrolytes Based on Anion‐Trapping Boron Moiety and Polyhedral Oligomeric Silsesquioxane for High Performance Lithium Metal Batteries
Source: Small Sci. 2024 Aug 12;4(10):2400183. doi: 10.1002/smsc.202400183 (PMC11934992; doi:10.1002/smsc.202400183)
Supplement: Supplementary file 1 — Supplementary Material [file SMSC-4-2400183-s001.pdf]

In Situ Formed Composite Polymer Electrolytes Based on Anion-Trapping Boron Moieties and Polyhedral Oligomeric Silsesquioxane for High Performance Lithium Metal Batteries

Chia-Chi Chang[a], Min-Hsien Shen[a], Yuan-Shuo Hsu[a], Hsisheng Teng[a,b,c] and Jeng-Shiung Jan[a,b,c]*

[a] Department of Chemical Engineering, National Cheng Kung University, Tainan 70101, Taiwan

[b] Hierarchical Green-Energy Materials (Hi-GEM) Research Center, National Cheng Kung University, Tainan 70101, Taiwan.

[c] Program on Smart and Sustainable Manufacturing, Academy of Innovative Semiconductor and Sustainable Manufacturing, National Cheng Kung University, Tainan, 70101, Taiwan

*Correspondence: Jeng-Shiung Jan, Department of Chemical Engineering, National Cheng Kung University, No. 1 University Road, Tainan City 701, Taiwan, E-mail: jsjan@mail.ncku.edu.tw, Phone: 886-6-275-7575 ext. 62660, Fax: 886-6-234-4496.

Table S1. The composition of the QSPEs and CPEs.

QSPEs/CPEs	Monomer (g) [a]			Plasticizer	Lithium salt
	AAPE	PEGMEMA	POSS	PEGDMA	LiTFSI
**A0**	0	2.0	---	1.6	1.2
**A10**	0.2	1.8	---	1.6	1.2
**A15**	0.3	1.7	---	1.6	1.2
**A20**	0.4	1.6	---	1.6	1.2
**A10P1**	0.2	1.8	0.02	1.6	1.2
**A10P3**	0.2	1.8	0.06	1.6	1.2
**A10P5**	0.2	1.8	0.1	1.6	1.2

[a] Initiator (AIBN) : 1 wt% of monomers

[b] Units : gram

Table S2. Glass transition temperature (T_g) of QSPEs and CPEs.

**QSPEs/CPEs**	**A0**	**A10**	**A15**	**A20**	**A10P1**	**A10P3**	**A10P5**
**T_g (°C)**	-56.5	-61.2	-58.8	-56.0	-54.7	-54.7	-52.6

Table S3. Summary of cycle performance of Li|CPE|LFP cells with other reported CPEs containing POSS.

Polymer electrolytes composition	Cycle number (retention)	Discharge capacity ($Li|LiFePO_4$)	Ref.
A10P1<br>AAPE/PEGMEMA/POSS/PEGDMA/LiTFSI	190 (100%)	143.2 mAh g^{-1}/0.2C (25°C)	This work
A10P3<br>AAPE/PEGMEMA/POSS/PEGDMA/LiTFSI	140 (100%)<br>220 (99.0%)	135.3 mAh g^{-1}/0.2C (25°C)<br>145.7 mAh g^{-1}/0.5C (60°C)	This work
POSS-g-PEGMEM/B-PEGMA SCE<br>POSS/PEGMEM/B-PEGMA/LiTFSA	100 (92.1%)<br>150 (>99%)	120.8 mAh g^{-1}/0.5C (25°C)<br>152.3 mAh g^{-1}/0.5C (60°C)	[21]
POSS-2PEG6K<br>POSS/PEG/LiTFSI	50 (>99%)<br>50 (>99%)	160 mAh g^{-1}/0.33C (90°C)<br>144 mAh g^{-1}/0.5C (90°C)	[12]
POSS-PIL-SPE-3<br>P[MAPOSS-VIM][TFSI]/ P(VDFHFP)/<br>[BMIM]TFSI/LiTFSI	100 (100%)	153 mAh g^{-1}/0.1C (26°C)	[40]
$PEGDA_{600}$-S-POSS<br>$PEGDA_{600}$/POSS-SH/$LiClO_4$	210 (>95%)<br>210 (>94%)	146 mAh g^{-1}/0.1C (60°C)<br>128 mAh g^{-1}/0.5C (60°C)	[41]
POSS-2PEGDA<br>PEO/PEGDA/POSS/LiTFSI	42 (92.7%)	138.6 mAh g^{-1}/0.1C (60°C)	[42]
$HTPE_{20}$<br>PCL20-PEG2K-PCL20/POSS-SH/$LiClO_4$	100 (>48.5%)	77.9 mAh g^{-1}/0.1C (60°C)	[43]

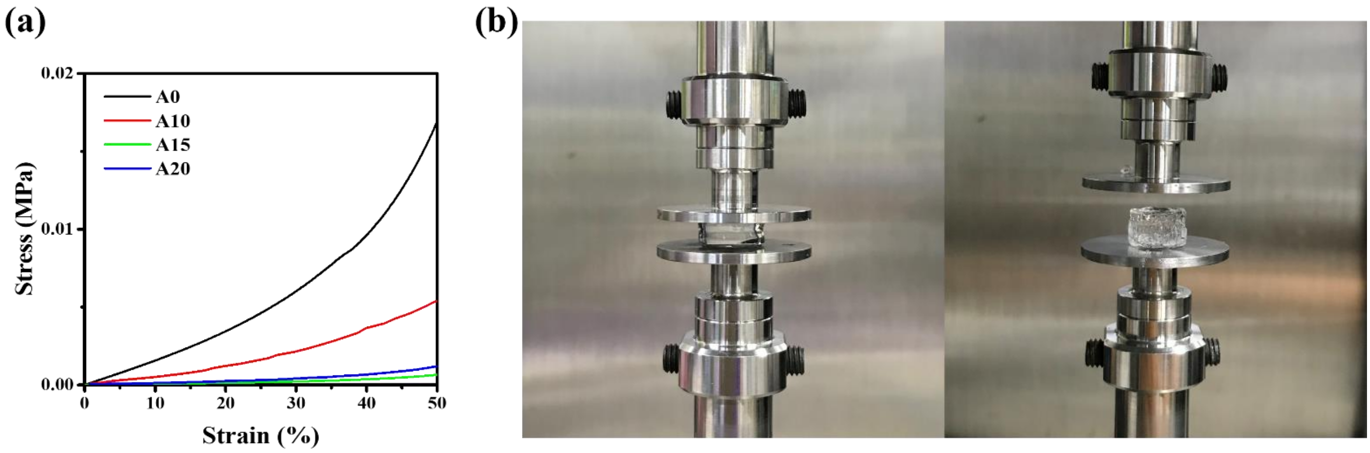

Fig. S1. (a) Compressive stress-strain curves of A-series QSPEs and (b) optical images of A10 QSPE with loading and unloading.

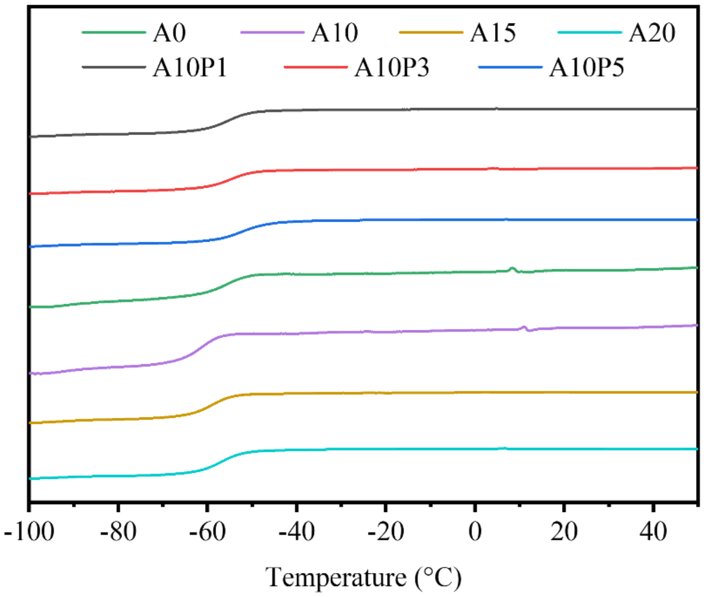

Fig. S2. DSC analysis of QSPE and CPE samples.

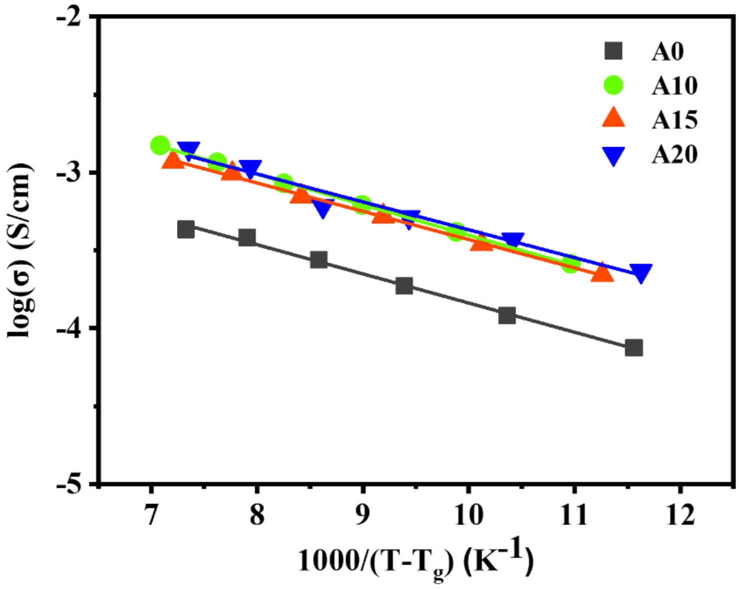

Fig. S3. Vogel-Tammann-Fulcher plot of ionic conductivity of A-series QSPEs.

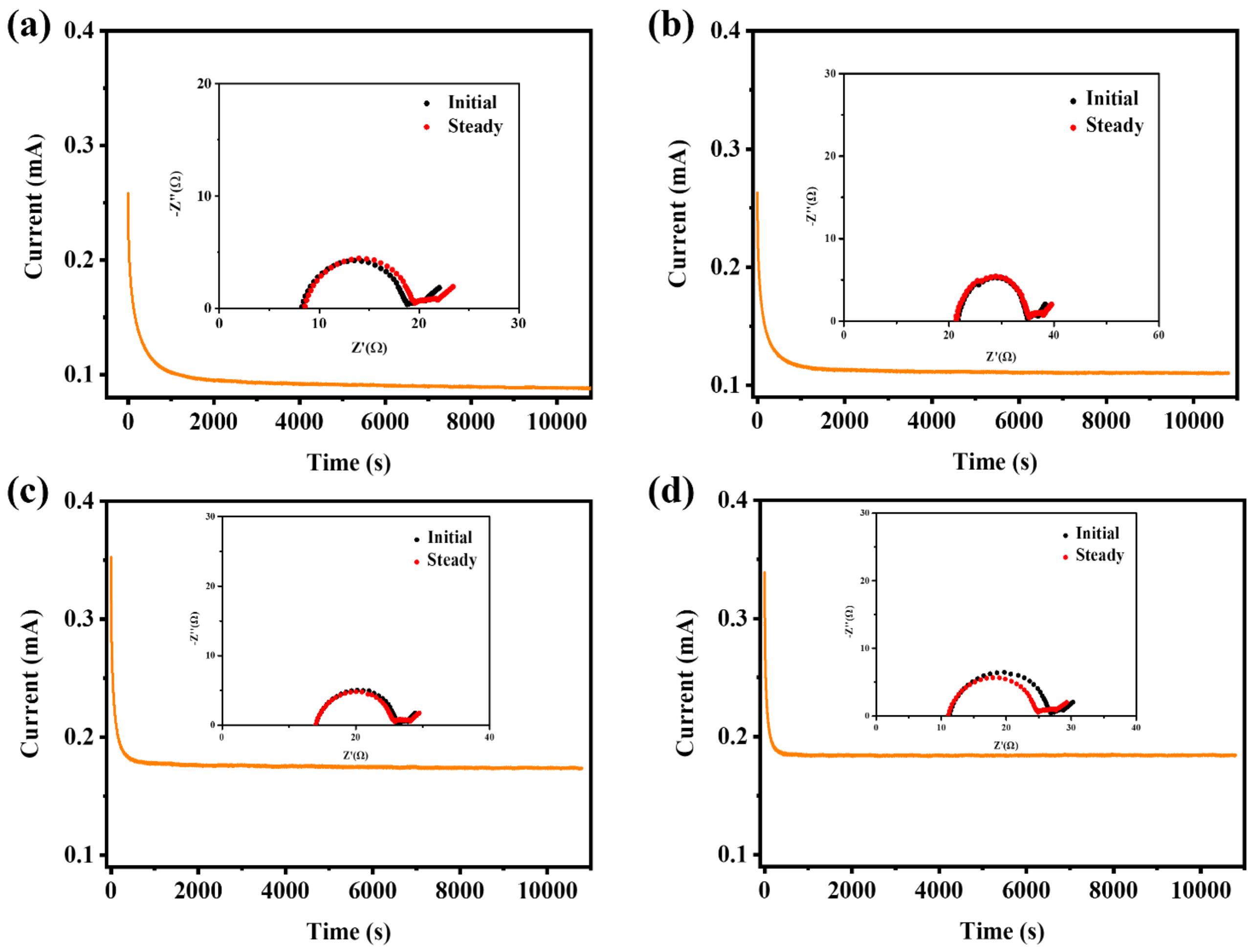

Fig. S4. Chronoamperometry profiles of (a) A0, (b) A10, (c) A15 and (d) A20 QSPEs.

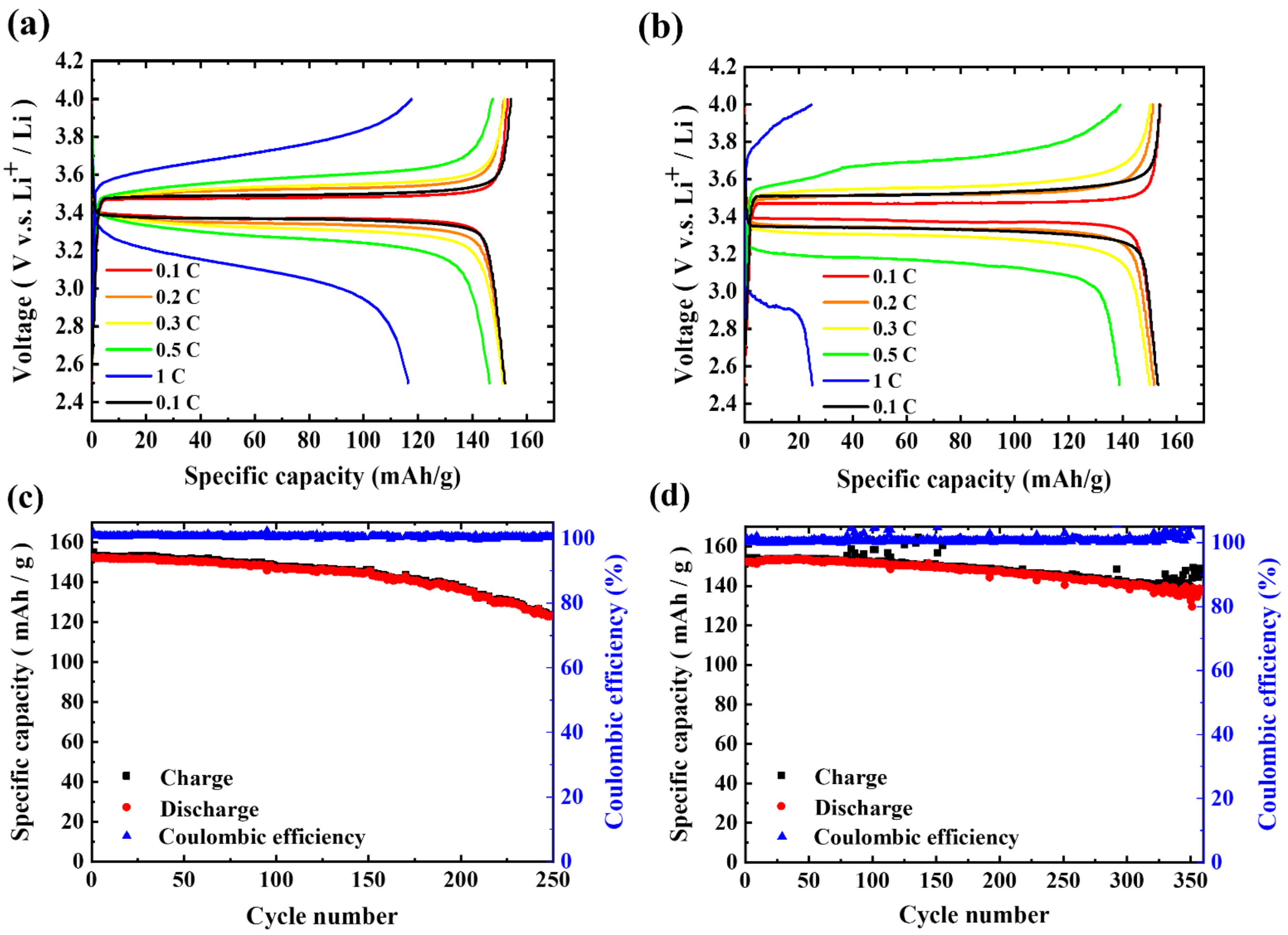

Fig. S5. (a, b) Charge and discharge curves and (c, d) cycling performance of (a, c) Li|A15|$LiFePO_4$ and (b, d) Li|A20|$LiFePO_4$ cells at 25 °C.

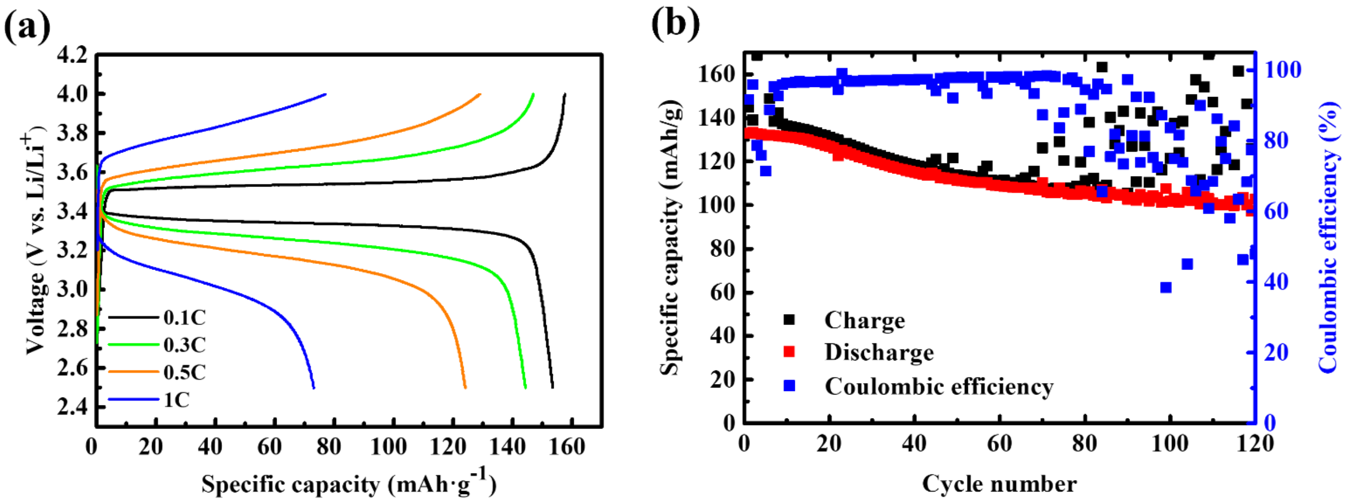

Fig. S6. (a) Charge and discharge curves and (b) cycling performance of Li|A0|$LiFePO_4$ cell at 25 °C.

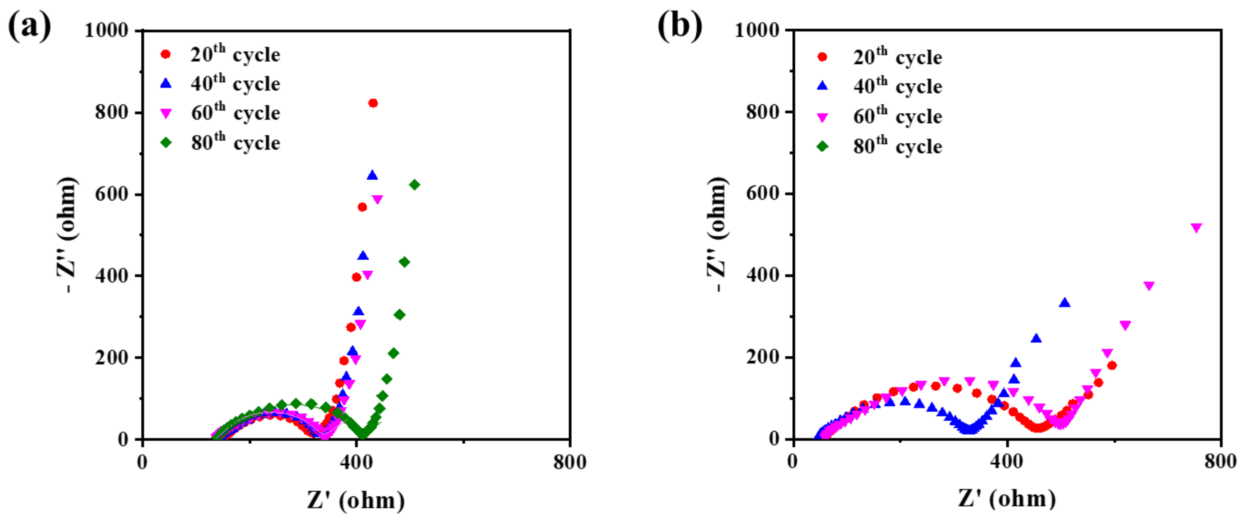

Fig. S7. EIS analysis of A10 membranes prepared via (a) *in-situ* (b) *ex-situ* thermal polymerized methods at different cycles.

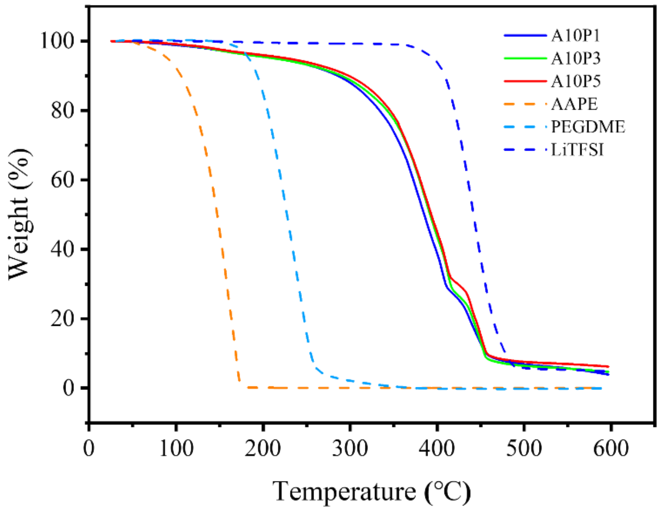

Fig. S8. TGA curves of AP-series CPE membranes, monomers, and lithium salt.

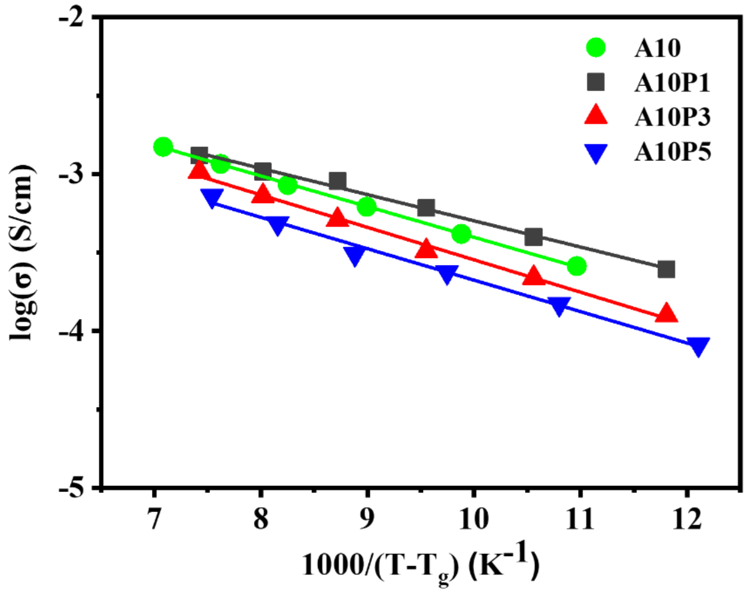

Fig. S9. Vogel-Tammann-Fulcher plot of ionic conductivity of AP-series CPEs.

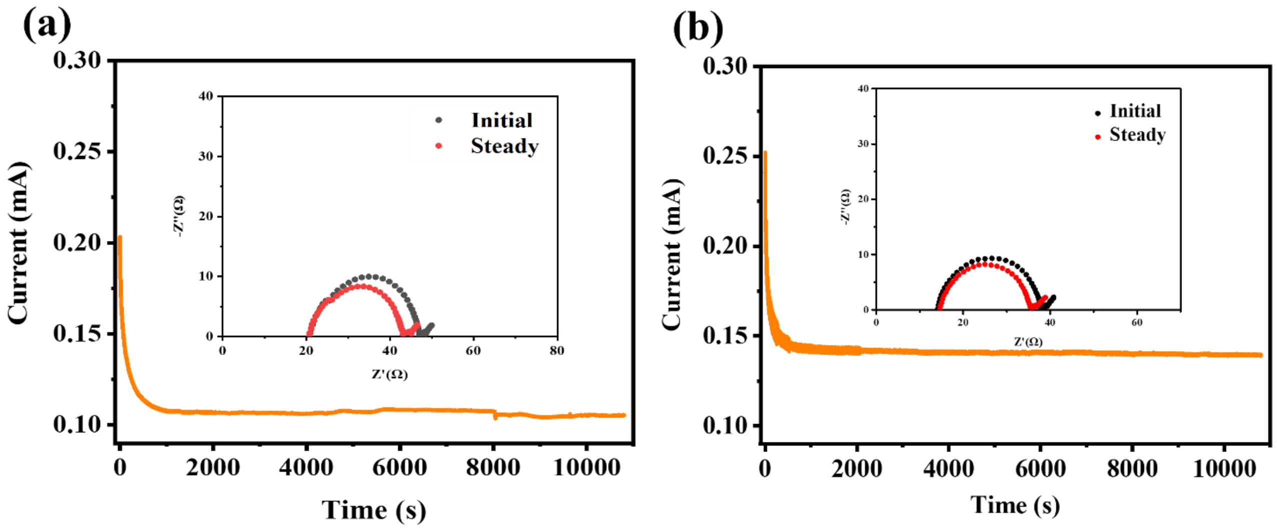

Fig. S10. Chronoamperometry profiles of (a) A10P1 and (b) A10P5 CPE membranes.

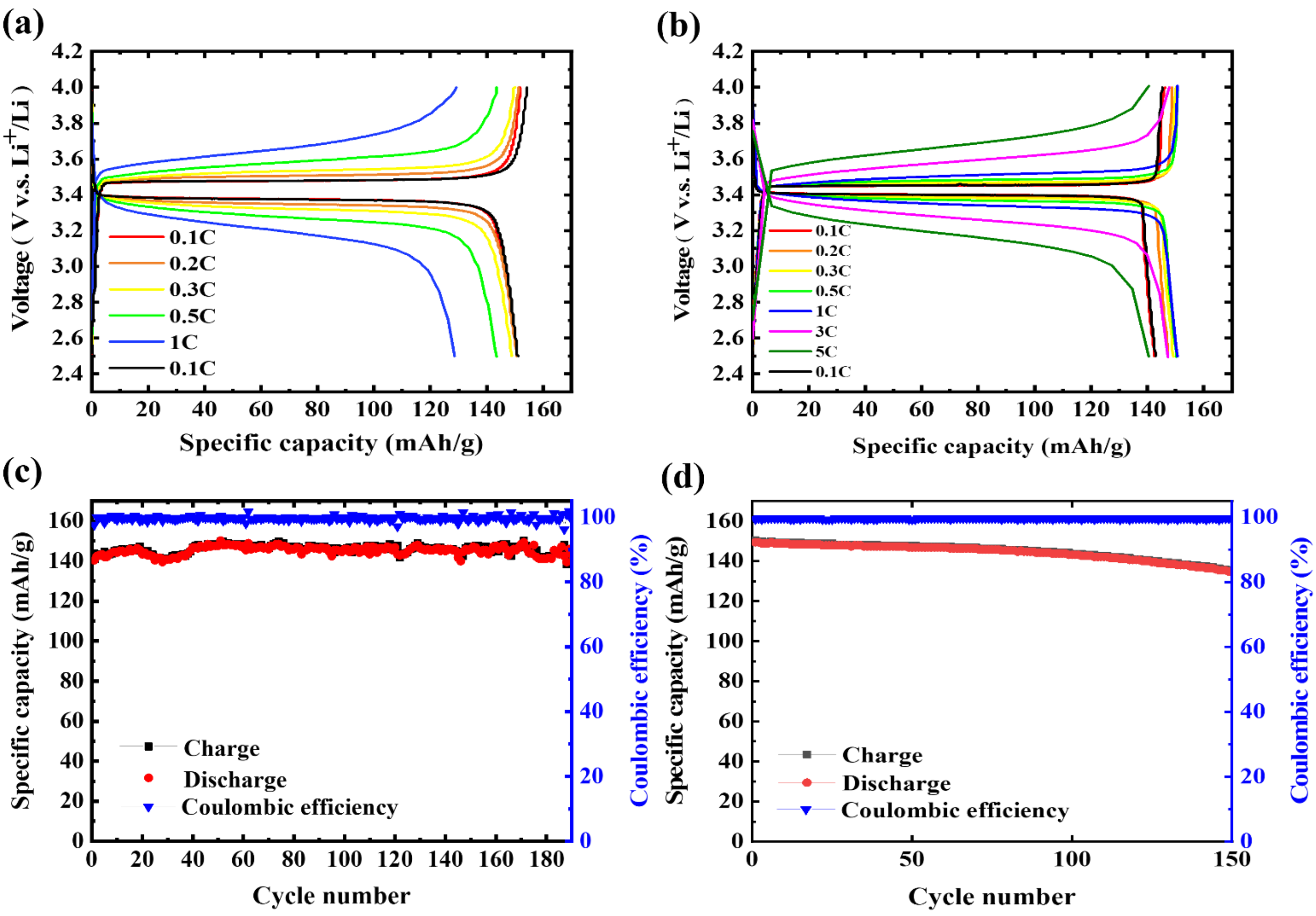

Fig. S11. (a, b) Charge and discharge curves and (c, d) cycling performance of Li|A10P1|LFP cell at (a, c) 25 and (b, d) 60 °C.

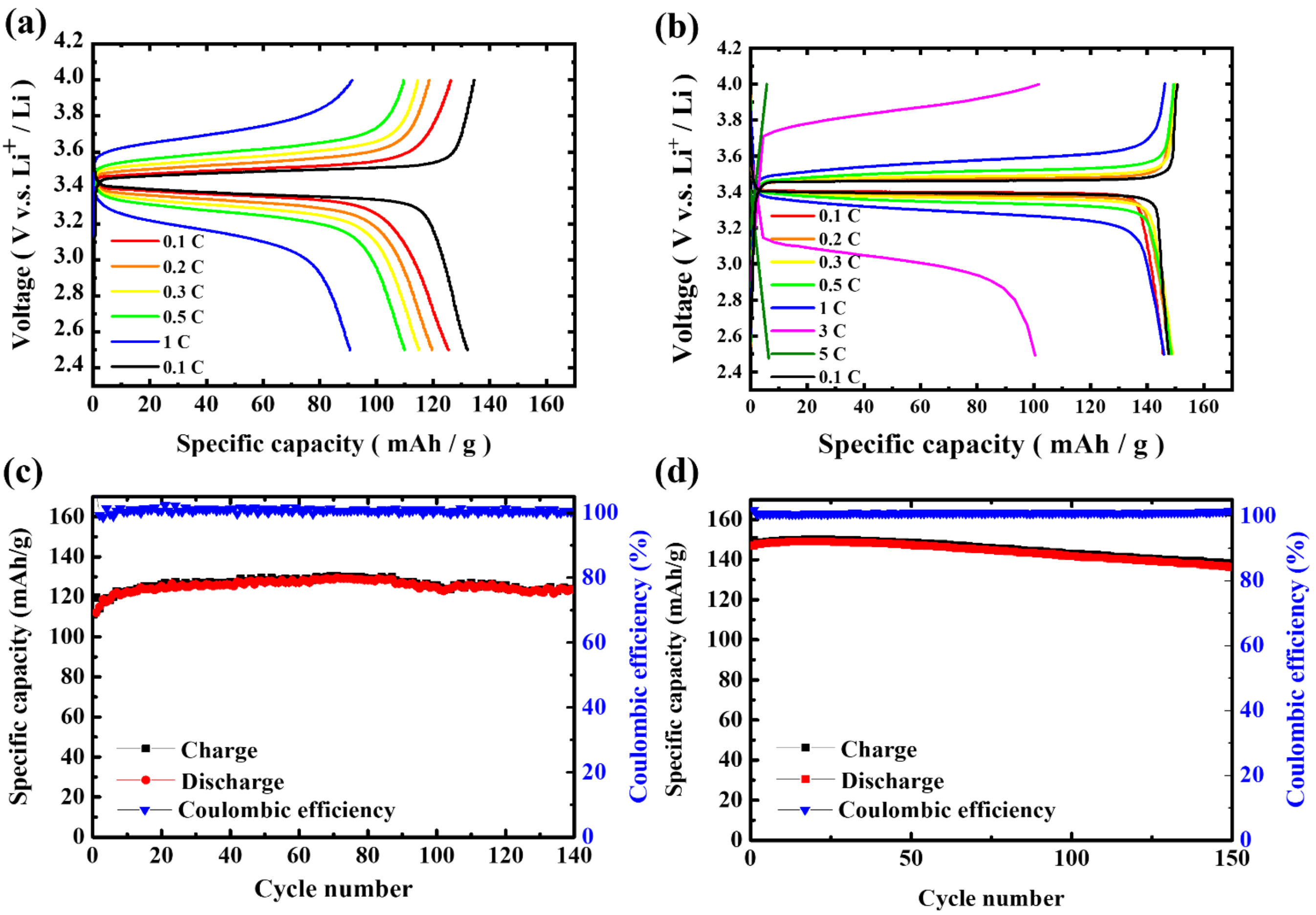

Fig. S12. (a, b) Charge and discharge curves and (c, d) cycling performance of Li|A10P5|LFP cell at (a, c) 25 and (b, d) 60 °C.

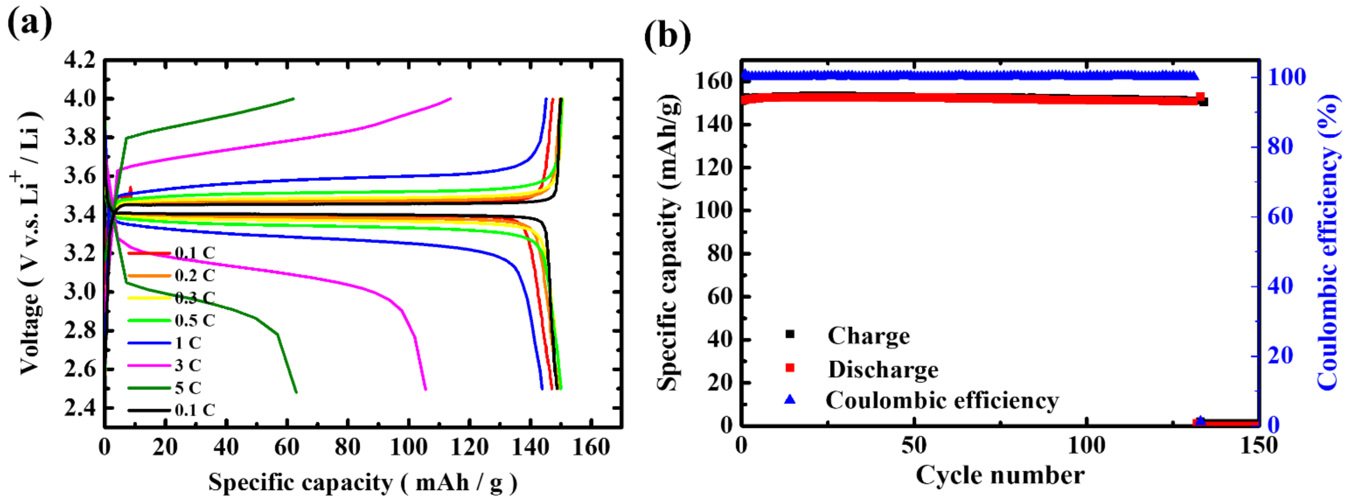

Fig. S13. (a) Charge and discharge curves and (b) cycling performance of Li|A10|LFP cell at 60 °C.

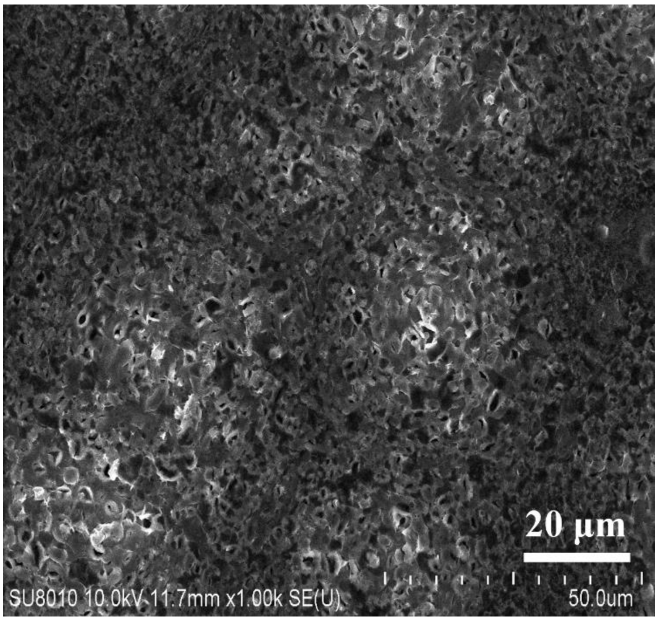

Fig. S14. SEM image of fresh lithium anode.
